# Technical note: Reduction of radiation dose using ultrasound guidance during transjugular intrahepatic portosystemic shunt procedure

**DOI:** 10.4103/0971-3026.76046

**Published:** 2011

**Authors:** Roshan S Livingstone, Shyamkumar N Keshava

**Affiliations:** Department of Radiology, Christian Medical College, Vellore - 632 004, India

**Keywords:** Radiation dose, TIPS, ultrasound

## Abstract

The transjugular intrahepatic portosystemic shunt (TIPS) procedure for decompression of the portal venous system generally performed under fluoroscopic guidance has undergone continuous technical modifications recently. Due to the length of the procedure, the fluoroscopy times are reasonably high, thus increasing the risk from ionizing radiation. Radiation doses were measured for 19 patients using dose area product (DAP) meter. The average DAP value for the TIPS procedure was 63.86 Gy cm^2^ (21.12-117.07). Radiation doses to patients can be reduced with the use of USG guidance and intermittent fluoroscopy screening.

## Introduction

The transjugular intrahepatic portosystemic shunt (TIPS) procedure for decompression of the portal venous system generally performed under fluoroscopic guidance has undergone continuous technical modifications in the current scenario.[1] One advance described in 2001 involves a direct intrahepatic portocaval shunt (DIPS) that entails an intravascular USG (IVUS)-guided puncture directly from the inferior vena cava (IVC) to the portal vein (PV).[2,3] A major advantage of USG guidance is the direct visualization of the needle track during puncture of the PV, eliminating the “blind” PV puncture during the standard TIPS technique. This potentially improves the safety and effectiveness of this procedure.[3]

## Materials and Methods

**Figure 1:**
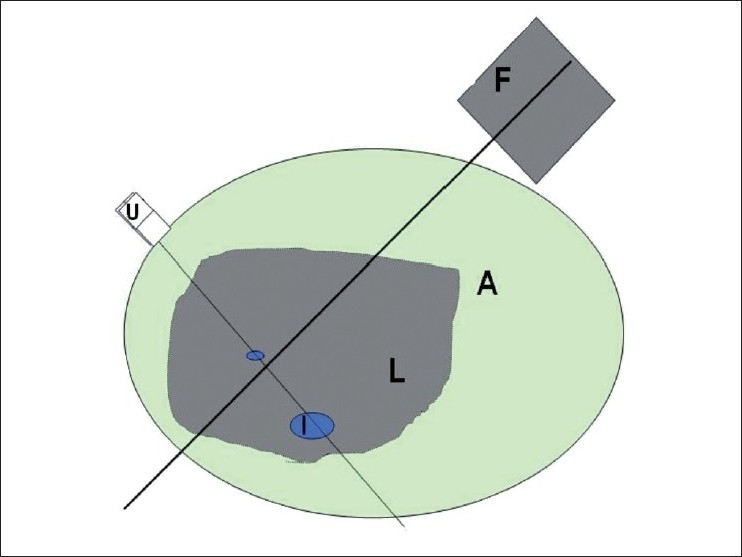
A line diagram shows the orientation of the ultrasound (U) transducer in the right antero-lateral aspect of the abdomen along the right branch of the portal vein and the inferior vena cava (I). The fluoroscopic (F) orientation is demonstrated in the left anterior oblique plane. A shrunken liver (L) surrounded by ascitic fluid (A) is represented

In our study, all TIPS procedures were performed using a DSA machine (Multistar, Siemens, Erlangen, Germany). Nineteen patients who underwent TIPS procedure were involved in the study. In 17 out of 19 patients, the shunt was created from the IVC to the portal vein and in 2 out of 19 patients, from the right hepatic vein to the portal vein. The USG transducer was kept on the right anterolateral aspect of the abdomen in an oblique sagittal plane to visualize both, the right branch of the portal vein and the IVC [Figure 1]. The fluoroscopic plane was almost perpendicular along the left anterior oblique plane [Figure 1]. Fluoroscopy screening was not performed while at the time of USG screening.

The average dose area product (DAP) measured using a DAP meter was 63.86 Gy cm^2^ (21.12–117.07). The average DAP contributions from fluoroscopy and image acquisitions were 48.06 Gy cm^2^ (5.28-96.59) and 15.8 Gy cm^2^ (2.13–63.91), respectively.

## Results and Discussion

In our center, TIPS and modified TIPS (cavoportal shunt/DIPS) are performed using fluoroscopy and abdominal USG guidance.[4] We believe that the use of USG guidance has a high success rate with minimal complications and a reduction in the length of the entire procedure. Apart from the clinical invasiveness of the procedure, TIPS is a procedure that produces high radiation doses as compared to other interventional procedures in the heart and skull,[5] with lengthy fluoroscopy times of the order of 30 min to 1 h or more depending upon the complexity of the study.[5] Zweers *et al*. have reported fluoroscopy times ranging from 9 to 115 min with total procedures lengths of 40–330 min.[6] Our study had an average fluoroscopy duration for the DIPS procedure of 19.2 min (4–32.1), leading to a notable reduction in the overall duration of the procedure. The doses reported in our study are significantly less as compared to those reported by Zweers *et al*., which ranged from 7 to 354 Gy cm^2^.[6]

In conclusion, the use of USG guidance with intermittent fluoroscopy can reduce the length of the procedure, fluoroscopy times, number of image acquisitions, and radiation doses imparted to patients while performing the TIPS procedure.
